# Low muscle mass and mortality risk later in life: A 10-year follow-up study

**DOI:** 10.1371/journal.pone.0271579

**Published:** 2022-07-28

**Authors:** Cristina Camargo Pereira, Valéria Pagotto, Cesar de Oliveira, Erika Aparecida Silveira

**Affiliations:** 1 Postgraduate Program in Health Sciences, Medical School, Federal University of Goiás (UFG), Goiania, Brazil; 2 Faculty of Nursing, Postgraduate Program in Nursing, Federal University of Goiás (UFG), Goiania, Brazil; 3 Department of Epidemiology & Public Health, University College London, London, United Kingdom; Ritsumeikan University, JAPAN

## Abstract

**Introduction:**

Little is known about the impact of low muscle mass (MM) assessed by calf circumference (CC), arm circumference (AC), arm muscle circumference (AMC), and corrected arm muscle circumference (CAMC)—on mortality risk later in life. We aimed to investigate the impact of low MM assessed by CC, AC, AMC and, CAMC on all-cause, cardiovascular, and cancer mortality risk.

**Methods:**

Data came from 418 older adults who participated in a 10-year follow-up prospective cohort study. Low MM was defined as a CC < 33 cm for women and < 34 cm for men and by the lowest tertile of AC, AMC, and CAMC stratified by sex. The log rank test, Kaplan-Meier curves, and Cox regression were used.

**Results:**

There were 147 deaths: 49 related to CVD and 22 to cancer. A small CC (HR = 1.57, 95% CI, 1.12–2.20), AMC (HR = 1.61, 95% CI, 1.13–2.30) and CAMC (HR = 1.45, 95% CI, 1.03–2.04) were associated with all-cause mortality. A small CAMC was a protective factor for CVD mortality (HR = 0.46, 95% CI, 0.22–0.98). In the Kaplan-Meier analysis, older adults with LMM presented low all-cause mortality survival, with AC (p < 0.05), AMC (p < 0.005), CAMC (p < 0.002), and CC (p < 0.001). Cancer mortality was associated with low CAMC (p < 0.020).

**Conclusions:**

Low MM assessed by anthropometric measures (AC, AMC, CAMC and CC) increased the all-cause mortality risk. A small CAMC decreased the CVD mortality.

## Introduction

The number of older adults is increasing rapidly globally, and by 2050 one in six individuals worldwide will be aged 65 and over [1]. Advancing age increases the occurrence of cardiovascular diseases (CVD), diabetes mellitus (DM), cancer, and respiratory diseases [2,3] which are the leading causes of death in this age group [2].

Prospective studies showed that typical health conditions in older adults such as functional disability, limited mobility, morbidity, and low physical performance have a significant impact on their mortality risk [4–9]. The health conditions that increase mortality risk are closely associated with reduced muscle mass (MM) that occurs with aging [10–12]. MM maintenance can decrease all-cause mortality risk and chronic diseases [10], improve sensitivity to insulin [10,13], increase energy expenditure [14], cardiorespiratory capacity and mobility in adults and older adults [15]. Therefore, MM seems to play an important role in the prevention and treatment of chronic diseases prevalent later in life such as CVD [16,17]. Its maintenance is also a strategy to minimize the adverse effects of chemotherapy such as fatigue, body composition, anxiety, strength and quality of life in people with cancer [12,18,19].

MM can be measured by different methods such as dual x-ray absorptiometry, bioelectrical impedance, ultrasound, computed tomography (CT), and magnetic resonance imaging (MRI) [12,19]. However, these methods are expensive and require some previous specific procedures that limit their use in the clinical context [20,21]. Considering that clinical practice scenarios, either public or private, represent the entryway to treatments or health care actions [22], it is important to use less costly, practical, and reproducible MM assessment methods, such as anthropometric measurements of arm circumference (AC) and calf circumference (CC) [23–27].

Small AC and CC have been previously associated with a lower survival rate in older institutionalized adults [28]. However, it is still unclear whether low MM, measured by anthropometric measurements, affects the all-cause, CVD, and cancer mortality risk in community-dwelling older adults. Therefore, the main aim of this study was to evaluate whether MM measured by different anthropometric measurements i.e. calf circumference (CC), arm circumference (AC), arm muscle circumference (AMC), and corrected arm muscle circumference (CAMC), impact on all-cause, CVD, and cancer mortality risk later in life using data from a ten-year follow-up cohort.

## Materials and methods

### Study population

The study was conducted in the city of Goiânia, capital of the state of Goiás, Midwestern Brazil. The procedures used in the Goiânia Older Adult Project cohort that started in 2008, were described in detail in previous publications [29–33]. The cohort population comprised of community-dwelling older adults, with a probabilistic sample representative of the municipality, aged 60 years or older (n = 418). All participants included in this research signed an informed consent form and authorized verification of the death certificate. This study was approved by the Research Ethics Committee of the Hospital das Clínicas of the Federal University of Goiás, Brazil (protocol number: 2.500.441).

## Follow-up

The participants were followed-up from baseline in 2008 until the date of the last interview held in 2018/2019.

### Mortality ascertainment

Mortality information was obtained from the Brazilian Mortality Information System from the baseline in 2008 to March 2019. All deaths were confirmed in home visits. The basic cause of death was coded using the World Health Organization International Classification of Diseases, Tenth Revision (ICD-10) [34]. CVD mortality was defined using the ICD-10 codes I100-I99, and mortality from cancer was defined using the ICD-10 codes C100-D48.

### Muscle mass (MM) assessment

MM was measured by the following anthropometric measurements: arm circumference (AC), arm muscle circumference (AMC), corrected arm muscle circumference (CAMC), and calf circumference (CC).

AC was measured at the intermediate point between the lateral projection of the scapular acromion process and the lower margin of the ulna olecranon with the person in an upright position [35]. The TSF was measured to calculate AMC and CAMC. The TSF measurement was performed with a Lange adipometer, with a constant pressure of 10 g/mm^2^ on the contact surface and an accuracy of 1 mm, with a 0–65 mm ruler. The measurement was taken on the back of the arm and midway between the point of the acromion and olecranon process while the arm was hanging relaxed. Three measurements were taken successively, and the average of three measurement was used [36]. CAMC was estimated using the formulas proposed by Heymsfield *et al*. [37] to determine muscle tissue reserve, correcting the bone area by sex. Calf circumference (CC) was measured at the point of greatest circumference in relation to the longitudinal line of the right calf [35].

Low MM was defined using the lowest tertile stratified by sex for AC (< 29.1 cm for men and 29.2 cm for women), AMC (< 23.1 cm^2^ for men and < 23.2 cm^2^ for women), and CAMC (< 20.1 cm^2^ for men and < 20.0 cm^2^ for women). For CC, a previously validated cut-off point was used for this same study population, with dual energy x-ray absorptiometry (DXA) data as the reference, with low MM values for CC being < 34 cm for men and < 33 cm for women [29].

The anthropometric measurements were performed by trained researchers, and validated according to the technique proposed by Habicht [38], with calculations of precision and accuracy verified by performing inter- and intra-technical error analyses to avoid measurement variability. All circumferences were measured twice using an inelastic tape (CESCORF) and averaged. All anthropometric measurements were collected by trained researchers using standardized procedures.

### Sociodemographic, lifestyle, and health variables

We also collected the following data: (i) sociodemographic characteristics (age, sex, skin colour, level of education, socioeconomic class, living with partner); (ii) health conditions, number of comorbidities and history of diseases previously diagnosed by physicians (diabetes and hypertension); (iii) lifestyle (physical activity level, smoking status, alcohol consumption and eating habits).

### Biomarkers and nutritional status

Weight (in kilograms) and height (in meters) were measured according to standard procedures [35]. Weight was measured using a calibrated portable digital electronic scale (Tanita) with a capacity of up to 150 kg and an accuracy of 100 g. Height was measured using a 2-meter tape measure, with an accuracy of 0.1 cm and fixed on a flat wall without a wall baseboard using a plumb line and a wooden square. Body Mass Index (BMI) was calculated by dividing the weight (kg) by the square of height (m). Systolic (SBP) and diastolic (DBP) blood pressures were obtained with a semi-automatic device (OMRON—HEM 705 CC) according to standardized recommendations [39]. Participants with a systolic blood pressure (SBP) ≥ 140 mmHg and/or diastolic (DBP) ≥ 90 mmHg or receiving pharmacological treatment for hypertension were considered hypertensive [39]. For the data on HDL-cholesterol, LDL-cholesterol, triglycerides-TG and glycemia, participants were asked to present the results of their laboratory blood tests performed up to three months before the interview date. Those reporting the use of hypoglycaemic drugs were classified as having diabetes mellitus. The drugs were identified according to medical prescription or possession and classified according to the Anatomical Therapeutic Chemical (ATC) Guidelines. The number of comorbidities was identified by answering the question “What diseases has your doctor said you have?”. The diseases mentioned were further categorized according to the ICD-10 [34].

### Statistical analysis

The anthropometric measurements were described according to the three mortality risk groups. The differences between the groups were evaluated using the Student’s t, Chi-square, or Fisher’s exact test.

The three outcomes, all-cause, CVD, and cancer mortality, were analysed according to the occurrence of low MM in 2008, measured by the following anthropometric variables: AC, AMC, CAMC, and CC, using three Cox regression models for adjustment, as follows: Model 1: sociodemographic variables; Model 2: Model 1 + lifestyle; Model 3: Model 1 + Model 2 + DM, hypertension, number of chronic diseases and biochemical markers (HDL-cholesterol, LDL-cholesterol, TG and glycemia).

The survival curves were plotted using the Kaplan-Meier’s method. The log rank test was used to compare the survival curves of older adults with low MM and adequate MM. For all statistical analyses, significance was determined at p <0.05 level. All analyses were performed using STATA 12.0 software. In cases of loss to follow-up, survival time was censored on October 10, 2018.

## Results

The sample characterization was as follows: mean age 70.69 ± 7 years (60 to 98 years), mean BMI = 26.97 ± 5.12 kg/m^2^, female (66%), white skin colour (46.4%), four years of education (41.2%), social class C (61%), living with a partner (45.2%), former smoker (43.3%), sedentary (35.2%), and not consuming fruits and vegetables on a daily basis (72.3%). The prevalence of diabetes was 23% and that of hypertension was 60% (Table 1). The baseline sample characteristics by mortality cause (all-cause, cardiovascular disease and cancer) are presented in Table 1.

**Table 1:** Baseline characteristics of participants stratified by all–cause, CVD and cancer mortality.

Variables	Baseline (n = 418)	All-cause (n = 147)	p	CVD (n = 49)	p	Cancer (n = 22)	p
**Gender, n (%)**			0.127^a^		0.636 ^a^		0.491^c^
Female	276 (66.0)	90 (32.6)		31 (11.4)		13 (4.7)	
Male	142 (33.9)	57 (40.2)		18 (12.9)		9 (6.5)	
**Age group, n (%)**			**0.000** ^a^		**0.000** ^a^		0.633 ^c^
60–69 years	203 (48.6)	45 (22.2)		15 (7.4)		9 (4.4)	
70–79 years	168 (40.2)	68 (40.5)		21 (12.8)		10 (6.1)	
80 years and over	47 (11.3)	34 (72.3)		13 (28.9)		3 (6.7)	
**Skin colour, n (%)**			0.379 ^a^		0.383 ^a^		0.740 ^c^
White	194 (46.4)	75 (38.7)		23 (11.9)		11 (5.7)	
Brown	178 (42.6)	57 (32.0)		18 (10.3)		8 (4.6)	
Black	46 (11.0)	15 (32.6)		8 (17.8)		3 (6.7)	
**Education level, n (%)**			**0.034** ^a^		0.289 ^a^		0.430 ^c^
Illiteracy	112 (29.9)	50 (44.6)		16 (14.7)		8 (7.3)	
1–4 years	154 (41.2)	52 (33.8)		18 (11.7)		9 (5.9)	
5–8 years	72 (19.3)	18 (25.0)		4 (5.6)		3 (4.2)	
9 years and over	36 (9.6)	10 (27.8)		5 (13.9)		0 (0.0)	
**Socioeconomic class, n (%)** *			**0.001** ^a^		**0.014** ^a^		0.654 ^c^
C	255 (61.0)	74 (29.0)		22 (8.7)		15 (5.9)	
D/E	159 (38.1)	72 (45.3)		26 (16.8)		17 (4.5)	
**Marital status, n (%)**			0.255 ^a^		0.999 ^a^		0.664 ^c^
Living without partner	189 (45.2)	72 (38.1)		22 (11.9)		11 (5.9)	
Living with partner	229 (54.8)	75 (32.7)		27 (11.9)		11 (4.8)	
**Smoking, n (%)**			0.163 ^a^		0.532 ^a^		0.333 ^c^
Never	198 (47.4)	62 (31.3)		20 (10.2)		9 (4.6)	
Current	39 (9.3)	18 (46.2)		6 (15.8)		4 (10.5)	
Former	181 (43.3)	67 (37.0)		23 (12.9)		9 (5.1)	
**Alcohol intake, n (%)**			0.889 ^a^		0.498 ^a^		1.000 ^c^
No	354 (84.7)	124 (35.0)		43 (12.4)		19 (5.5)	
Yes	64 (15.3)	23 (35.9)		6 (9.4)		3 (4.7)	
**Physical activity, n (%)**			**0.000** ^a^		**0.002** ^a^		0.901 ^c^
Sedentary	147 (35.2)	75 (51.0)		29 (20.3)		8 (5.6)	
Irregularly active	184 (44.0)	46 (25.0)		13 (7.1)		9 (4.9)	
Active	84 (20.1)	25 (29.7)		7 (8.4)		5 (6.0)	
Very active	3 (0.7)	1 (33.3)		0 (0.0)		0 (0.0)	
**Consumption of fruits and vegetables**			0.523 ^a^		0.405		0.807 ^c^
No	302 (72.3)	109 (36.1)		38 (12.7)		17 (5.7)	
Yes	116 (27.7)	38 (32.7)		11 (9.7)		5 (4.4)	
**Chronic Diseases, n (%)**							
Diabetes mellitus			**0.011** ^a^		**0.020** ^a^		0.796 ^c^
No	320 (76.6)	102 (31.8)		31 (9.8)		18 (5.7)	
Yes	98 (23.4)	45 (30.6)		18 (18.6)		4 (4.1)	
Hypertension			0.333 ^a^		0.208 ^a^		0.509 ^c^
No	166 (39.7)	63 (37.9)		15 (9.4)		10 (6.3)	
Yes	252 (60.3)	84 (33.3)		34 (13.5)		12 (4.7)	
**Number of diseases, n (%)**			0.439 ^a^		0.052 ^a^		0.120 ^c^
None	22 (5.3)	5 (22.7)		0 (0.0)		0 (0.0)	
1–2	214 (51.2)	78 (36.5)		21 (9.9)		16 (7.6)	
3 and over	182 (43.5)	64 (35.2)		28 (15.6)		6 (3.3)	
**HDL-cholesterol (mg/dL), mean (±SD)**	47.88 (±12.34)	46.35 (±11.33)	0.204^b^	45.52 (±9.46)	0.289 ^b^	43.1 (±8.73)	0.168 ^b^
**LDL-cholesterol (mg/dL), mean (±SD)**	119.06 (±45.65)	119.44 (±45.20)	0.933 ^b^	109.91 (±42.45)	0.281 ^b^	111.89 (±30.29)	0.597 ^b^
**TG (mg/dL), (mean) (±SD)**	154.25 (±84.68)	155.54 (±92.05)	0.875 ^b^	144.77 (±89.28)	0.514 ^b^	203.67 (±125.41)	**0.038** ^b^
**Glucose (mg/dL), (mean) (±SD)**	103.09 (±40.13)	103.85 (±38.57)	0.852 ^b^	99.1 (±32.99)	0.559 ^b^	103.36 (±31.72)	0.981 ^b^
**Nutritional status (BMI kg/m^2^)**							
Continuous, (mean) (±SD)	26.97 (±5.12)	25.77 (±4.92)	**0.000** ^b^	27.14 (±4.6)	0.833 ^b^	25.94 (±3.97)	0.322 ^b^
Underweight (<18.49 kg/m^2^), n (%)	17 (4.1)	11 (64.71)	**0.013** ^a^	2 (12.5)	0.875 ^a^	0 (0.0)	0.396 ^c^
Normal (18.5–24.99 kg/m^2^), n (%)	135 (32.3)	52 (38.52)		14 (10.4)		11 (8.2)	
Overweight (>25–29.99 kg/m^2^), n (%)	153 (36.3)	54 (35.29)		20 (13.5)		6 (4.1)	
Obese (≥30 kg/m^2^)	113 (27.0)	30 (26.55)		13 (11.5)		5 (4.4)	

BMI: Body mass index; CDV: Cardiovascular disease; HDL: High-density lipoprotein; LDL: Low-density lipoprotein; SD: Standard deviation; TG: Total triglycerides.

^a^ x^2^ test.

^b^ Student’s t-test.

^c^ Fisher’s Exact Test.

*Class A and B were excluded from the analysis due to presence of few individuals.

During the ten-years follow-up, 147 (35.2%) deaths occurred. Of these, 49 (33.3%) died of CVD and 22 (14.9%) of cancer. The all-cause mortality rates were statistically different for low MM values evaluated by CC (p < 0.000), AC (p < 0.027), AMC (p < 0.004), and CAMC (p < 0.002) and the cancer mortality rates were statistically different considering CAMC (p < 0.0036) (Table 2).

**Table 2:** Anthropometric measurements of participants at baseline stratified by all-cause mortality, CVD and cancer.

Variables	Baseline (n = 418)	All-cause (n = 147)	p	CVD (n = 49)	p	Cancer (n = 22)	p
**Calf circumference (CC)**							
Continuous, (average) (±DP)	34.33 (±3.44)	33.31 (±3.55)	**<0.001** ^**b**^	34.30 (±3.12)	0.934 ^**b**^	34.04 (±3.68)	0.672 ^**b**^
Normal, n (%)	265 (63.7)	76 (28.7)	**<0.001** ^**a**^	29 (11.1)	0.619 ^**a**^	12 (4.6)	0.370 ^**c**^
Low, n (%)	151 (36.3)	70 (46.4)		19 (12.7)		10 (6.7)	
**Arm circumference (AC)**							
Continuous, (average) (±DP)	30.68 (±4.37)	29.34 (±4.40)	**<0.001** ^**b**^	30.67 (±3.91)	0.941 ^**b**^	29.72 (3.83)	0.276 ^**b**^
Normal, n (%)	271 (64.8)	85 (31.4)	**0.027** ^**a**^	34 (12.8)	0.452 ^**a**^	11 (4.2)	0.170†
Low, n (%)	147 (35.2)	62 (42.2)		15 (10.3)		11 (7.5)	
**Arm muscle circumference (AMC)**							
Continuous, (average) (±DP)	24.37 (±3.12)	23.73 (±3.29)	**0.002** ^**b**^	24.26 (±2.90)	0.767 ^**b**^	24.37 (±2.84)	0.984 ^**b**^
Normal, n (%)	278 (66.7)	84 (30.2)	**0.004** ^**a**^	29 (10.6)	0.328 ^**a**^	14 (5.1)	0.817 ^**c**^
Low, n (%)	139 (33.3)	62 (44.6)		19 (13.8)		8 (5.8)	
**Corrected arm muscle circumference (CAMC)**							
Continuous, (average) (±DP)	21.81 (±4.08)	20.54 (±4.10)	**<0.001** ^**b**^	21.76 (±3.55)	0.895 ^**b**^	20.94 (±3.74)	0.294 ^**b**^
Normal, n (%)	278 (66.7)	83 (29.8)	**0.002** ^**a**^	36 (13.1)	0.205 ^**a**^	10 (3.6)	**0.036** ^**c**^
Low, n (%)	139 (33.3)	63 (45.3)		12 (8.8)		12 (8.8)	

CVD: Cardiovascular diseases; n: Number; SD: Standard deviation.

^**a**^ X2 test

^**b**^Student’s t test

^c^Fisher’s exact test.

In the Cox regression analysis, low MM measured by CC (HR = 1.84; 95% CI, 1.32–2.54), AMC (HR = 1.59; CI95%: 1.15–2.22), and CAMC (HR = 1.67; 95% CI, 1.20–2.32) significantly increased all-cause mortality risk in the ten-year follow-up. Low MM measured by CAMC increased cancer mortality (HR = 2.60; 955 CI, 1.12–6.03) (Table 3).

**Table 3:** Cox’s crude proportional risk analysis to assess associations of low muscle according to anthropometric measures with all-cause mortality, CVD and cancer.

Low muscle mass	All causes (n = 147)		CVD (n = 49)		Cancer (n = 22)	
	HR (95%CI)	p	HR (95%CI)	p	HR (95%CI)	p
**Calf circumference (CC)** ^**a**^	1.84 (1.32–2.54)	**<0.001**	1.31 (0.73–2.33)	0.368	1.66 (0.72–3.85)	0.236
**Arm circumference (AC)** ^**b**^	1.38 (0.99–1.92)	0.052	0.83 (0.45–1.52)	0.547	1.91 (0.83–4.42)	0.128
**Arm muscle circumference (AMC)** ^**c**^	1.59 (1.15–2.22)	**0.005**	1.42 (0.79–2.54)	0.234	1.24 (0.52–2.96)	0.625
**Corrected arm muscle circumference (CAMC)** ^**d**^	1.67 (1.20–2.32)	**0.002**	0.74 (0.38–1.42)	0.366	2.60 (1.12–6.03)	**0.026**

HR: Hazard Ratio; CI: Confidence interval; CVD: Cardiovascular diseases.

Cutoff points.

^a^Calf circumference (CC): <34 cm for men and <33 cm for women.

^b^Arm circumference (AC): <29.1 cm for men and <29.2 cm for women.

^c^Arm muscle circumference (AMC): <23.1 cm^2^ for men and <23.2 cm^2^ for women.

^d^Corrected arm muscle circumference (CAMC): <20.1 cm^2^ for men and <20.0 cm^2^ for women.

In the adjusted Cox regression analysis, all-cause mortality risk was significantly associated with the three low MM anthropometric measurements i.e. CC (HR = 1.57; 95% CI, 1.12–2.20), AMC (HR = 1.61; 95% CI, 1.13–2.30), and CAMC (HR = 1.45; 95% CI, 1.03–2.04) (Table 4). A small CAMC was associated with a lower CVD mortality risk (HR = 0.46, 95% CI, 0.22–0.98). There was no significant association for the other low MM parameters (Table 5).

**Table 4:** Fully adjusted Cox proportional risk analysis to assess the association of low muscle mass according to anthropometric measures with all-cause mortality (n = 147).

Low muscle mass	Model 1^a^	Model 2^b^	Model 3^c^
	HR (95%CI)	HR (95%CI)	HR (95%CI)
**Calf circumference (CC)**	1.54 (1.10–2.14)*	**1.46 (1.05–2.05)** *	**1.57 (1.12–2.20)** *
**Arm circumference (AC)**	1.15 (0.83–1.62)	1.18 (0.85–1.66)	1.23 (0.87–1.72)
**Arm muscle circumference (AMC)**	1.53 (1.07–2.18)*	**1.58 (1.11–2.25)** *	**1.61 (1.13–2.30)** *
**Corrected arm muscle circumference (CAMC)**	1.38 (0.98–1.95)	**1.46 (1.04–2.05)** *	**1.45 (1.03–2.04)** *

HR: Hazard Ratio; CI: Confidence Interval.

Adjustment.

^a^Model 1: Sociodemographic (age, sex, skin colour, education, socioeconomic class, marital status).

^b^Model 2: Model 1 + lifestyle (smoking, alcohol consumption, physical activity, consumption of fruits and vegetables).

^c^Model 3: Model 2 + Chronic Diseases, Number of diseases and biochemical markers (HDL-cholesterol, LDL-cholesterol, TG and serum glucose).

* p <0.05.

**Table 5:** Fully adjusted to Cox’s proportional risk to assess associations of low muscle according to anthropometric measures with CVD mortality (n = 49) and Cancer (n = 22).

Low muscle mass	Model 1^a^		Model 2^b^		Model 3^c^	
	CVD	Cancer	CVD	Cancer	CVD	Cancer
	HR (95%CI)	HR (95%CI)	HR (95%CI)	HR (95%CI)	HR (95%CI)	HR (95%CI)
**Calf circumference (CC)**	1.23 (0.68–2.23)	2.18 (0.87–5.45)	1.15 (0.64–2.08)	2.05 (0.79–5.35)	1.29 (0.72–2.34)	1.40 (0.42–4.68)
**Arm circumference (AC)**	0.64 (0.34–1.19)	1.88 (0.76–4.63)	0.81 (0.44–1.49)	1.75 (0.68–4.45)	0.88 (0.48–1.64)	1.01 (0.27–3.84)
**Arm muscle circumference (AMC)**	1.44 (0.80–2.58)	1.46 (0.54–3.94)	1.36 (0.76–2.43)	1.46 (0.52–4.10)	1.41 (0.78–2.54)	0.52 (0.11–2.38)
**Corrected arm muscle circumference (CAMC)**	**0.39 (0.18–0.85)** *	2.36 (0.89–6.21)	**0.41 (0.19–0.87)** *	2.20 (0.82–5.94)	**0.46 (0.22–0.98)** *	2.76 (0.79–9.51)

HR: Hazard Ratio; CI: Confidence Interval; CVD: Cardiovascular diseases.

Adjustment.

^a^Model 1: Sociodemographic (age, sex, skin colour, education, socioeconomic class, marital status).

^b^Model 2: Model 1 + lifestyle (smoking, alcohol consumption, physical activity, consumption of fruits and vegetables).

^c^Model 3: Model 2 + Chronic Diseases, Number of diseases and biochemical markers (HDL-cholesterol, LDL-cholesterol, TG and serum glucose.

* p <0.05.

**Fig 1:**
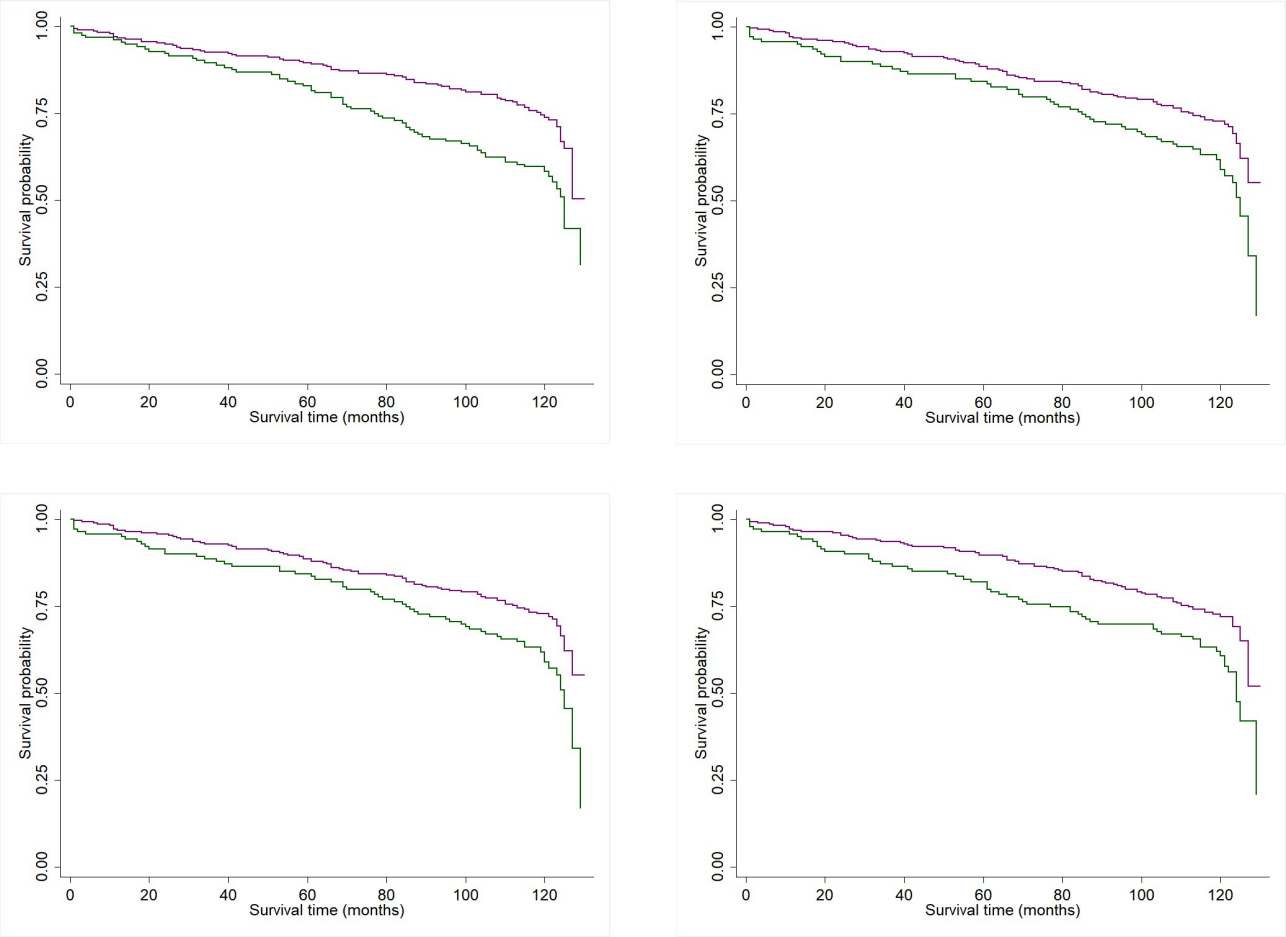
Survival curves of the population studied according to the anthropometric measurements (— Normal; — Low). (A) Normal Calf circumference; Low Calf circumference. (B) Normal Mid-Arm circumference; Low Mid-Arm circumference. (C) Normal Mid-Arm muscle circumference; Low Mid-Arm muscle circumference. (D) Normal Corrected arm muscle circumference; Low Corrected arm muscle circumference.

For all-cause mortality, the log rank test showed that participants with low MM measured by all anthropometric measurements (CC: p < 0.001; AC: p < 0.05; AMC: p < 0.005; CAMC: p < 0.002;) had shorter survival times compared to participants with adequate MM (Fig 1A, 1B, 1C and 1D).

The survival curves for CVD in participants with low and normal MM measured by AC (p = 0.546), AMC (p = 0.231), CAMC (p = 0.364), and CC (p = 0.366) showed no significant differences. With regards to cancer mortality risk, a small CAMC (p < 0.020) reduced survival. Small AC (p = 0.122), AMC (p = 0.625), and CC (p = 0.231) did not change cancer survival.

## Discussion

To the best of our knowledge, this study was the first to analyse the impact of low muscle mass measured by various anthropometric measures on all-cause, CVD, and cancer mortality risk in community-dwelling older adults from a Latin American community. This study used four anthropometric measurements to evaluate muscle mass and our findings showed that having a small AC, AMC, CAMC, and CC increased significantly the all-cause mortality risk after 10 years of follow-up. In addition, older adults with a small CAMC had a shorter survival time for cancer and lower risk of death from CVD.

### Low muscle mass and all-cause mortality

This study showed that the lowest AMC and CAMC tertiles and small CC increased the risk of death. The results from the survival curves analyses showed that individuals with low muscle mass measured by AC, AMC, CAMC, and CC have lower survival rates after a ten-year follow-up. These results corroborate previous studies [40–53] in older community-dwelling adults that evaluated the impact of low muscle mass assessed by anthropometric measurements on mortality risk. However, these studies were mostly conducted in European and Asian countries. A study [52] conducted with 1,298 fragile older Mexican adults showed that a CC < 31 cm was a risk factor for mortality after a 14-year follow-up. Findings from the Longitudinal Aging Study Amsterdam (LASA) cohort with community-dwelling individuals aged 65 or older showed that a AC < 30 cm increased the risk of death in men by 1.8 times and in women by up to 2.3 times after a 15-year follow-up [47]. Another study, using data from community-dwelling older Japanese adults, showed that a small AMC was a risk factor for mortality after a two-year follow-up [48]. In British older adults, higher AMC values were associated with a reduction of up to 15% in all-cause mortality after a 15-year follow-up [40].

Interestingly, in a community-dwelling sample aged 70 and older, a European study [51] showed that CAMC was not associated with all-cause mortality after six years, but a study in Australia [52] showed that a CAMC lower than ≤ 21.4 cm^2^ in men and ≤ 21.6 cm^2^ in women increased the risk of death after an eight-year follow-up. Data from a cohort with a six-month follow-up showed that the risk of death has been reduced by up to 5% for each unit of increased CAMC [42].

Decreased anthropometric measurements may indicate low muscle mass, being associated with worse health conditions and a higher likelihood of death [54,55]. Therefore, the use of anthropometric measures is relevant to evaluate and monitor older adults in health settings where the most effective methods to evaluate body composition are expensive and difficult to access [30].

The most used sophisticated methods to assess body composition, such as dual x-ray absorptiometry and bioelectrical impedance, despite having a good precision in the measurement of body compartments, are not routinely used in clinical practice, due to the need for trained personnel, high costs and time spent for their performance [12,19]. Anthropometric measurements are valid alternatives for the assessment of body composition, since they are relatively fast, inexpensive and a large number of people can be examined in a short period of time [23–27]. In addition, they can be widely used to estimate body composition in several clinical conditions, such as cardiovascular disease, cancer and sarcopenia later in life.

### Low muscle mass and CVD mortality

Previous findings on the association between anthropometric measures and cause-specific mortality in older adults living in the community are limited [50,51]. Furthermore, the available evidence from the studies [49–51,56] that evaluated the impact of muscle mass on CVD and cancer mortality using anthropometric measures remains inconclusive.

The findings of the present study showed that a small CAMC reduces the risk of CVD mortality by 54%. Most previous investigations on the impact of low muscle mass on CVD mortality used only AMC measurement [49,50,56]. One study evaluating 1,061 older European adults living in the community aged 70 to 77 years showed that CAMC was not associated with CVD mortality [51]. However, in this same study, increased AC was associated with a higher risk of CVD mortality [51].

Data from the Bangladesh Health Effects of Arsenic Longitudinal Study (HEALS) cohort, which followed 1,975 individuals aged 18 and older [41] for 8 years showed that a small AC was a risk factor for CVD in people with low BMI. In the NHANES III, with 11,958 people aged 20 to 90 years, small AC was a risk factor for CVD death after a 14-year follow-up [49]. In the Charleston Heart Study [56], low AC was a risk factor for CVD mortality in black men, but not in white men.

Since the CAMC calculation involves triceps skinfold (DCT) and AC with bone mass correction, the decrease in CAMC probably reflects a gain in subcutaneous fat mass. The associations of each body fat deposit in the risk of CVD vary, since the upper and lower parts of the body contain divergent fat deposits with different biological functions [56].

Regional differences in the severity of adipose inflammation, storage and renewal of lipids, release of adipokines and endocrine effects are among the mechanisms potentially responsible for the aforementioned proposed associations [56–58].

Even for similar types of fat, subcutaneous adipose tissue in the arm was considered less susceptible to unregulated release of free fatty acids compared to abdominal subcutaneous adipose tissue [59]. A study on postmenopausal older women showed that trunk fat was associated with an increased risk of CVD, while fat in the extremities (arms and legs) was not [56].

On the other hand, CAMC is considered a surrogate marker to indicate the muscle mass used to determine a muscle tissue reserve, reflecting the sarcopenia that is associated with mortality in the older adults [46]. The mechanism underlying the relationship of sarcopenia with all-cause mortality in community-dwelling older adults is not fully elucidated [10]. However, sarcopenia is closely associated with physical function, conferring greater risk to fractures, disability, dependence, recurrent hospitalization and mortality [12]. Therefore, considering changes in body composition with ageing it is important to evaluate the changes using different anthropometric measurements to predict mortality.

### Low muscle mass and cancer mortality

In the present study, the ten-year survival was lower in individuals with low muscle mass compared to those with normal muscle mass using CAMC. To date, there are no studies on the association between CAMC and the risk of death from cancer in older adults living in the community. A study evaluating cancer mortality in obese and non-obese people in the US used AC to measure muscle mass and reported no association between AC and cancer mortality risk [45].

The relationship between low muscle mass and cancer mortality later in life also remains uncertain. A greater understanding of the underlying mechanisms of muscle loss is needed. However, it is known that maintaining muscle mass can improve the metabolism and increase energy reserves, increasing, consequently, the chances of older adults coping with disease [60]. Therefore, low muscle mass estimated by anthropometric measurements may constitute indicators of poor prognosis in the face of cancer diagnosis at advanced ages.

This study demonstrates that anthropometric measures such as CC, AMC and CAMC used as indicators of low muscle mass can predict mortality in community-dwelling older adults. Knowing that CC is more affected by edema than AC [61] and considering that TSF and AC data are accessible and easy to measure in community-dwelling older adults, we recommend the use of AC and TSF in the nutritional assessment to calculate AMC and CAMC.

Overall, there are no consistent results between anthropometric measurements and the risk of death from specific causes. It should be noted that most of the studies were conducted on young adults in European countries.

The most important limitation of this study include the lack of muscle strength measurement and tests that evaluate muscle function (such as usual gait speed). It is recognized that strength is better than mass in predicting adverse outcomes, particularly mortality [12]. Another potential limitation of this study relates to its low statistical power to analyse the association with cancer mortality due to the number of deaths from this cause in the follow-up period. This study has strengths such as the analysis of non-institutionalized or hospitalized older adults. Most of the evidence on the studied topic comes from these groups. Another positive aspect is the ten-year follow-up period, especially in Brazil. Finally, this study used a validated CC cut-off point [25] in the same population, in which the DXA was used as reference.

These results have relevant implications for clinical practice in gerontology and geriatrics, as well as for planning public health actions. The measurement of these anthropometric measures should be incorporated into health care practices for older adults, as they are more practical and cheaper than other methods to measure muscle mass and, consequently, helping the development of mortality prevention measures. Anthropometric evaluations are effective as the first step in screening older patients to identify those most at risk of death and to target interventions to prevent loss of muscle mass. The early detection of low muscle mass can reduce disability and, in turn, increase the survival rate later in life. In additional, future studies should investigate the relationship of these anthropometric measurements by CVD type and primary site of cancer.

## Conclusion

Low muscle mass evaluated by the anthropometric measurements i.e. AC, AMC, CAMC and CC increased the all-cause mortality risk in older community-dwelling adults but not for cancer and CVD mortality. Except for a small CAMC, which increased the risk of cancer mortality and reduced the risk of CVD mortality.

## Supporting information

S1 File (XLSX)

S2 File (DOCX)

## Abbreviations

CC: calf circumference
AC: arm circumference
AMC: arm muscle circumference
CAMC: corrected arm muscle circumference
K-M: Kaplan-Meier
MM: Muscle Mass
