# Supplementary material for: Low muscle mass and mortality risk later in life: A 10-year follow-up study
Source: PLoS One. 2022 Jul 28;17(7):e0271579. doi: 10.1371/journal.pone.0271579 (PMC9333286; doi:10.1371/journal.pone.0271579)
Supplement: S2 File — (DOCX) [file pone.0271579.s002.docx]

**Dictionary of variables.**

| **Variable name** | **code** |
| --- | --- |
| **Gender** | sexo08 |
| Female | 1 |
| Male | 2 |
| **Age** | idade08 |
| **Skin colour** | cor08 |
| White | 1 |
| Brown | 2 |
| Black | 3 |
| **Years of study** | anoest08 |
| **Socioeconomic class** | classesocial |
| A | A |
| B | B |
| C | C |
| D | D |
| **Marital status** |  |
| Living without partner | 0 |
| Living with partner | 1 |
| **Smoking** | fuma08 |
| Never | 0 |
| Current | 1 |
| Former | 2 |
| **Alcohol intake** | bebalc08 |
| No | 0 |
| Yes | 1 |
| **Physical activity** | atfisica08 |
| No | 0 |
| Yes | 1 |
| [**Consumption**](https://www.collinsdictionary.com/pt/dictionary/english-portuguese/consumption) [**of**](https://www.collinsdictionary.com/pt/dictionary/english-portuguese/of) [**fruits**](https://www.collinsdictionary.com/pt/dictionary/english-portuguese/fruit) [**and**](https://www.collinsdictionary.com/pt/dictionary/english-portuguese/and) [**vegetables**](https://www.collinsdictionary.com/pt/dictionary/english-portuguese/vegetable) | flv08 |
| No | 0 |
| Yes | 1 |
| Diabetes mellitus | ddia08 |
| No | 0 |
| Yes | 1 |
| Hypertension | dha08 |
| No | 0 |
| Yes | 1 |
| **Number of diseases** | numorbi08 |
| None | 0 |
| 1 - 2 | 1 |
| 3 and over | 2 |
| **HDL-cholesterol** | exhdl08 |
| **LDL-cholesterol** |  |
| **Triglycerides** | exldl08 |
| **Glucose** | exglij08 |
| **Body Mass Index (BMI kg/m²)** | imc08 |
| **Calf circumference (CC)** | cp08 |
| **Arm circumference (AC)** | cb08 |
| **Arm muscle circumference (AMC)** | cmb08 |
| **Corrected arm muscle circumference (CAMC)** | cama08 |
| **All-cause mortality** | obito |
| **CVD mortality** | dcv |
| **Cancer mortality** | cancer |
| **Time of follow-up** | time |
